# Clinical preparedness programme as perceived by first‐year diagnostic radiography students in South Africa

**DOI:** 10.1002/jmrs.740

**Published:** 2023-11-09

**Authors:** Heidi Thomas, Gerhardus George Visser Koch

**Affiliations:** ^1^ Department of Medical Imaging and Therapeutic Sciences Cape Peninsula University of Technology Cape Town South Africa

**Keywords:** Clinical environment, diagnostic radiography, student readiness, student transition

## Abstract

**Introduction:**

There is an increasing emphasis on exploring ways to improve students' transition from the classroom into the clinical environment. Diagnostic radiography (DR) students undergo rigorous theoretical and practical training before they are exposed to the clinical environment. It has been found that when DR students commence clinical learning in the workplace for the very first time, they experience difficulties in this transition. At the site of investigation, a newly integrated and dedicated clinical preparedness (CP) programme was offered; however, little is known about the DR students' perceptions of this programme.

**Methods:**

A qualitative approach coupled with a self‐developed, cross‐sectional research tool was employed. First‐year DR students were purposefully sampled against the inclusion and exclusion criteria. The principle of voluntary participation was upheld throughout the data collection process. The data were analysed using Braun and Clarke's six steps of thematic analysis.

**Results:**

Forty‐two responses were collected. Following a thematic analysis, two overarching themes were developed, namely: (1) reflections on the CP programme and (2) suggestions for future CP programmes.

**Conclusion:**

This study demonstrated the need for effective CP programmes as an approach to ease the transition of first‐year DR students from the classroom into the clinical environment. Several suggestions, for example, an extended CP programme and a clinical information pack were made for the offering of similar programmes in the future.

## Graphical abstract

This study sought to investigate first‐year diagnostic radiography students' perceptions of a newly developed clinical preparedness (CP) programme. Based on the gathered responses, recommendations were developed intended for radiography educators wanting to introduce CP programmes into their curricula.

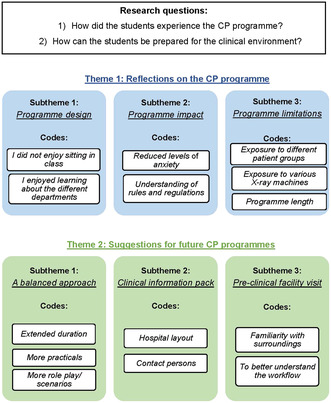

## Introduction

Diagnostic radiography (DR) is a specialised skill that relies heavily on clinical competence.^1^ DR students undergo rigorous theoretical, practical, and clinical training before they graduate as competent healthcare professionals. The practical and clinical training is also known as work‐integrated learning (WIL).^2^ The International Journal of Work‐integrated Learning^3^ describes WIL as an educational approach whereby a student is placed into a real‐life environment and given an opportunity to practise what they have been taught. Govender et al.^4^ agree with this description of WIL and further add that WIL is imperative for developing the necessary graduate attributes for the real world of work.

It is of utmost importance that students be adequately prepared for the clinical environment. Both authors, in their capacity as full‐time educators involved in the undergraduate training of first‐year students in a four‐year Bachelor of Science (BSc) degree programme, are of the opinion that a strong foundation phase is critical. This foundation phase can be seen as the student's first year of studies, which paves the way towards the student's overall success. At the same time, it is important to keep in mind that most first‐year students have often just completed their school leaving certificates. In view of this, most students may not have had the opportunity to experience a professional and/or clinical environment or workspace.

The literature reports that student radiographers experience significant challenges when they transition from the classroom to the clinical environment. Factors hindering the positive integration of students into the clinical environment include feelings of uncertainty, fear of the unknown, anxiety, and stress.^5^, ^6^ The concept *“clinical preparedness”* (CP) is acknowledged and introduced in this paper as a critical component in the undergraduate curriculum to integrate students into the clinical education environment. Clinical preparedness is defined as “*the ability to develop critical skills, integrate theory with practice, apply problem‐solving skills, develop interpersonal skills and become socialised into the formal and informal norms, protocols and expectations*”.^7^ The establishment of effective CP programmes particularly for first‐year DR students thus becomes indispensable for equipping students with the necessary skills, knowledge, and confidence to navigate the challenges of the complex healthcare environment in which they will find themselves. In South Africa (SA), a paucity of published literature prevails over the CP of DR students and no specific guidelines were found from the professional society (Society of Radiographers of South Africa) or governing board (Health Professions Council of South Africa). The purpose of this study was, therefore, to explore the DR students' perceptions of the CP programme as it relates to their readiness for the clinical environment.

### The Clinical Preparedness (CP) Programme

As part of undergraduate training requirements in SA, students are placed for WIL at public or private clinical training facilities approved by the Health Professions Council of South Africa (HPCSA). At the research site, first‐year students were placed at a single facility without rotating to other sites during their first year of studies.

The CP programme under evaluation was first introduced in 2022 of which the aim was to provide a solid foundation for fostering professionalism, practical skills, and patient‐centred care. The CP programme was incorporated serving as a dedicated offering, supplementary to the standard theoretical classes and practical tutorials. The motivation behind the programme's incorporation stemmed from informal discussions between the authors and supervising clinical radiographers responsible for guiding students during their clinical training. These discussions underscored the necessity for an exclusive and concentrated CP programme, to augment the students' preparedness for the clinical environment. The programme was carefully designed by the authors based on their personal reflections and teaching experiences.

A three‐day CP programme was deemed compulsory for all first‐year DR students as an in‐person event. The authors, with the assistance of a clinical instructor employed at the research site, facilitated the offering of this programme. The CP programme was structured to include several topics and activities. This intervention encompassed the facilitation of supplementary lectures, discussions, practical demonstrations in the skills laboratory and role‐play scenarios. The topics and activities were designed to include themes relevant to, effective communication, peer interaction, the picture archiving and communication system, logbook orientation, image critique, and radiation protection. Table 1 depicts some of the components of the CP programme under review.

**Table 1:** Components embedded in the CP programme.

Day	Components
One	➔ Small group activities (introductions, role play, etc.) ➔ Instructional videos on personal protective equipment ➔ Questions and answers sessions
Two	➔ Theoretical lectures ➔ Logbook and timesheet discussions ➔ X‐ray room orientation ➔ Practical demonstrations ➔ Questions and answers sessions
Three	➔ Hands‐on practice sessions (equipment handling and patient positioning technique) ➔ Questions and answers sessions

## Methods

### Population and sample

The study population included all 64 first‐year actively registered, DR students from a single research site in SA. A purposive sampling technique was deemed appropriate for this study. Purposive sampling, by definition, allows qualitative researchers to recruit participants who can provide in‐depth and detailed information about a phenomenon under investigation.^8^ Thus, first‐year DR students were purposefully selected after having attended the CP programme and after having already transitioned into the clinical environment. This selection stems from the rationale that these students were already exposed to the CP programme and the clinical environment and would be able to best express their perceptions following their attendance and participation.

### Data collection

Qualitative data were collected through a self‐administered, open‐ended questionnaire. Data collection commenced after students completed the final clinical placement of their first academic year. The questionnaires were completed via an online platform using Google Forms. The online questionnaire was used to gain an understanding of first‐year DR students' experience of the CP programme and how it can be improved going forward. Therefore, the questionnaire consisted of open‐ended questions to gain meaningful insights from participants. The questionnaire consisted of six open‐ended questions and two closed‐ended demographic questions (refer to Table 2).

**Table 2:** Questionnaire.

Type	Questions
Open‐ended	➔ The CP programme prepared me well enough/not well enough for what I was exposed to during my clinical rotations. Please explain your answer ➔ What did you enjoy most about the CP programme? Please explain your answer ➔ What did you enjoy least about the CP programme? Please explain your answer ➔ What would you have liked to be exposed to during the CP programme? Please explain your answer ➔ In your opinion, what would you suggest the facilitators do differently to prepare next year's group of first‐year students for their clinical rotations? ➔ Please add any additional comments you might have about the CP programme
Closed‐ended	➔ I attended the CP programme (yes/no) ➔ I completed my clinical training in a (government hospital/private practice)

An independent recruiter assisted with the recruitment process. This individual had no direct involvement in the student's clinical training and thus eliminated potential power imbalances. Participation in the study was completely voluntary which was explained to participants in a study information leaflet. A total of forty‐two online questionnaires were completed. In keeping with qualitative research, data were collected, downloaded from Google Forms, and reviewed continuously until saturation was reached. This means that data collection ceased when little or no new useful information relative to the study objectives emerged.^9^

During this study, bracketing was prioritised. Therefore, the researchers had set aside (bracketed) their own feelings, preconceived ideas, and opinions by regularly engaging in debriefing sessions with a senior lecturer at the same research site so that potential bias during data collection, analysis and interpretation be avoided.^9^

### Data analysis

All the completed online questionnaires were exported to an Excel spreadsheet. Analysis of the data was guided by Braun and Clarke's six steps of thematic analysis.^10^ The six steps involved: (1) familiarising oneself with the data, (2) generating initial codes, (3) searching for themes, (4) reviewing themes, (5) refining and naming themes, and (6) producing a report.

**Figure 1:**
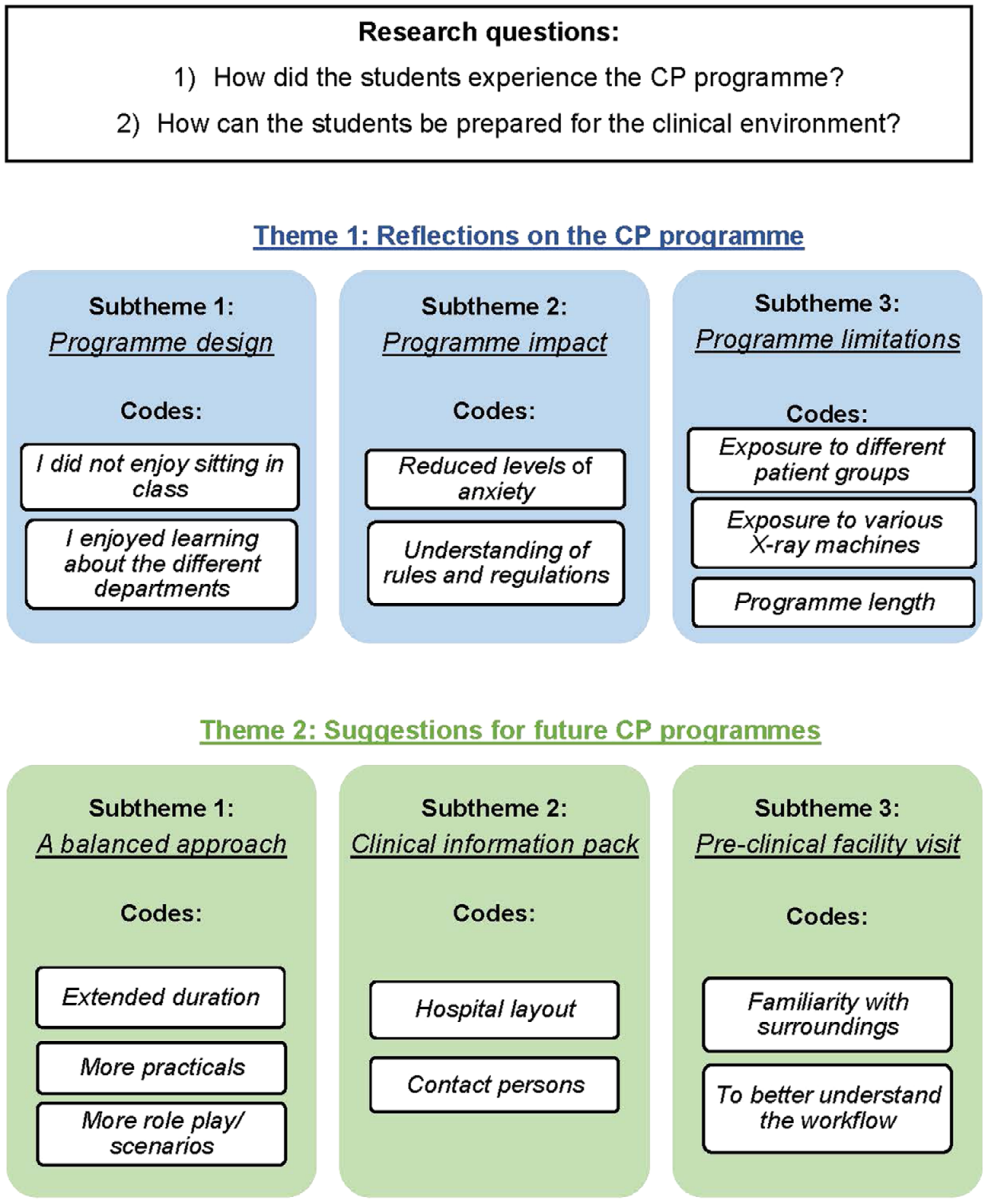
The data analysis process.

To gain a deep and clear understanding of the data the researchers read and re‐read the data as it appeared on the Excel spreadsheet. The researchers did independent, manual coding by highlighting interesting parts in the data using highlighters to indicate patterns in the data. The codes were compared before the researchers jointly searched for themes. This was followed by a review of the themes. A thematic map to demonstrate how codes were grouped together in order to form potential themes guided the process. The next step required the researchers to define and name the themes in an inductive and interpretive manner. The last step required the researchers to finalise the analysis process and to produce a report for publication. Figure 1 depicts the data analysis process.

### Trustworthiness

The trustworthiness principles introduced by Guba and Lincoln,^11^ were incorporated into this study. These principles are: (1) credibility, (2) transferability, (3) dependability, (4) confirmability, and (5) authenticity. *Credibility* was maintained by triangulation. Sources for triangulation included the data from the questionnaires, the researchers' reflective notes, and discussion points. Credibility was further ensured through member checking. To ensure transferability, a detailed write‐up of the research methodology is available. Furthermore, a dense description of the research context and participants are provided.^12^ The researchers ensured d*ependability* by keeping detailed notes of the research process from start to finish. To maintain *confirmability*, copies of the data sheets and the researchers' debriefing notes are kept.^13^ In keeping with authenticity, verbatim quotes to support the developed themes and subthemes are provided.

### Ethical considerations

Ethical clearance was sought and obtained from the Faculty of Health and Wellness Sciences, Research Ethics Committee (FHWSREC), Cape Peninsula University of Technology (HWS‐REC 2022/H22). Consent to participate in this research was obtained prior to participants completing the online questionnaire. Voluntary participation was adhered to, and the principle of diminished autonomy was maintained throughout the research consent process. Anonymity was ensured by omitting any personal identifiers of participants and the research site. Data were stored on a password‐protected computer that could only be accessed by the principal researcher. Data were downloaded and shared between researchers via password‐protected emails.

## Results

Participants completing clinical training in the public healthcare sector (*n* = 33) and private healthcare sector (*n* = 9) responded to the questionnaire. The data analysis provided invaluable insights into the first‐year DR students' perceptions of the CP programme. Following the data analysis process (Fig. 1), the following themes were identified: (1) reflections on the CP programme and (2) suggestions for future CP programmes. The subthemes will be discussed in this section along with the verbatim quotations from the study participants.

### Theme 1: Reflections on the CP programme

This theme describes the overall reflections of the programme as experienced by the participants. Three subthemes were developed by the authors following a critical evaluation of the programme, namely: (1) programme design, (2) programme impact, and (3) programme limitations.

#### Subtheme 1: Programme design

Participants' responses indicated a great emphasis on the overall experience of the CP programme as it relates to the structure and content of the CP programme. Responses relevant to the structure of the programme hinted at some presentations being too long and that too much information was covered.

The lecturers really tried to inform us as well as possible, the presentations they put together were good, but maybe a bit too long to stay focused for. (P3)

I did not enjoy sitting in class the whole day and not doing enough practical things. (P17)

It was too much information. (P25)

Responses relevant to the content indicate that the practical and interactive approach to learning such as interacting with peers and facilitators, engaging in role play, and practising in the skills laboratory were highly favoured. Learning about injuries and the various departments was also preferred. Students indicated a need for more practice on the X‐ray equipment.

I enjoyed learning about injuries. (P5)

I enjoyed practising on the X‐ray machine in the skills lab, putting the theory I was taught in class into practice. I also enjoyed interacting with fellow classmates and getting to understand the role of teamwork in healthcare. (P9)

I enjoyed the demonstration videos regarding our positioning techniques, and I also think that the interaction with everyone was fun. (P11)

I enjoyed the engagement between us and the facilitators. The programme was more interactive, and the facilitators allowed us to ask as many questions as we wanted. (P12)

I enjoyed the part where we did act, poems and all the fun things. (P15)

I enjoyed learning about the different departments within the hospital because I wasn't aware that there were so many different departments. (P32)

If I am being honest, I would have appreciated a bit more exposure on handling the equipment as it took some getting used to when we started our clinicals, but because it is incorporated in the clinical environment, we were able to get more comfortable with machine handling over time. (P7)

#### Subtheme 2: Programme impact

Participants expressed various views relevant to the impact of the programme on their preparedness for the clinical environment. Most of the responses indicated that students felt prepared for the clinical environment following exposure to the CP programme. They suggested that the CP programme prepared them mentally and physically for the workflow, how to act in emergency situations, how to deal with patients, and introduced them to rules and regulations pertaining to the workplace. One student described feeling less anxious following exposure to the CP programme. The responses below depicted participants' readiness.

The programme prepared me well enough for what I was supposed to be doing, with workflow and how to treat patients and the people I am working with. (P1)

I learnt how to be cautious during clinicals and be able to act well in case of emergency. I also got mentally prepared for the things I would see in the hospital. (P5)

I had a gap year in 2021, and I feel like I was not really prepared for anything going into this course. I really enjoyed preparedness week as it was informative, and it was fun at the same time. Lots of effort was made to make us as comfortable as we could be going into the clinical environment, thus I was less anxious after the programme. (P11)

Prepared me well enough, it was very informative and helped me mentally and physically. (P25)

It prepared me well enough as when I got to the department, I had a clue of what to do and what was expected of me as a student radiographer. (P31)

The preparedness programme prepared me well enough as it taught me the rules and regulations that need to be followed while in the clinical environment. It also taught me what is expected of me to be able to do and what I need to know for my year level. (P33)

#### Subtheme 3: Programme limitations

A few gaps in the offering of the CP programme were identified from the participants' responses. The three‐day programme offered was considered too short for comprehensive and thorough coverage of required information. Participants believed the programme needed to include a focus on different patient interactions and exposure to various X‐ray equipment.

I didn't enjoy that it was such a short program as I think that we needed more knowledge to prepare ourselves. (P32)

I think it could have been better if the program continued for 2 weeks. They should have trained us well by going in‐depth with different kinds of patients that students will see in the workplace and maybe have scenarios. (P10)

To learn about the different patients, we will get and the different machines we will be working with. (P28)

Have people play roles of different patients, with different levels of seriousness and age. (P10)

A few examples of the diversity and uniqueness of patients we can encounter, although this was to be expected I feel like when I was faced with this challenge during the clinical setting, I was a bit flustered. (P11)

### Theme 2: Suggestions for future CP programmes

Although the participants, in general, found the CP programme to be useful in preparing them for the clinical environment, a few suggestions for the future offerings of a similar programme were made. The suggestions were based on the participants' experience of the programme and their own, personal experience having already transitioned into their respective clinical environments. Three subthemes were identified namely: (1) a balanced approach, (2) clinical information pack, and (3) pre‐clinical facility visit.

#### Subtheme 1: A balanced approach

From the suggestions, a need for a more balanced approach to the CP programme was deemed critical for aiding the students' readiness for the clinical environment. A more balanced programme, as per the participants, should include (1) an extended programme, longer than three days, (2) more hands‐on, practical simulation sessions, (3) the inclusion of more real‐life scenarios to help them prepare psychologically for what they may be exposed to in the clinical environment, and (4) to include shorter and less theoretical presentations.

Extend the days that we have the workplace preparedness. (P15)

I think it could have been better if the program continued for 2 weeks. (P10)

I least enjoy doing all of the writing work instead of practical. (P28)

I did not enjoy sitting in class the whole day and not doing enough practical things. (P17)

I think more time should be allocated for the chest and abdomen tutorials and remove unnecessary activities. (P9)

I feel that the lecturers could've given us more scenarios as to what to expect in the working environment as well as what to do in the first few days of clinical. (P14)

The lecturers really tried to inform us as well as possible, the presentations they put together were good, but maybe a bit too long to stay focused for. (P3)

The short amount of time, maybe it can be a little bit longer. It was also too much information. (P25)

The long lectures. It was difficult to stay focused and it was too much information to write down as they went through it very fast. (P3)

#### Subtheme 2: Clinical information pack

An information booklet was recommended, whereby a visual map of the hospital layout should be included as well as general information about the department, for example, contact persons and contact details. Some participants felt disorientated by their new surroundings.

I would have liked more information regarding my hospital and a hospital plan, I felt lost because I did not know where the wards were, and I did not know who to ask. (P25)

The most important thing is to prepare students so the programme should involve more of the hospital environment. (P10)

#### Subtheme 3: Pre‐clinical facility visit

A pre‐clinical rotation site visit to the students' respective clinical departments before commencing with the rotations was highlighted as a suggestion. It is believed that future pre‐exposure visits will ease the students' transition into this new and unknown environment and, to witness, first‐hand, the workflow of the clinical department.

I think facilitators should take students to the hospital at least one day of the three days and educate them based on what they see. (P12)

Do the tour of the hospital more than once so that the new students are more comfortable walking in the hospital and will not get lost when they are sent somewhere. (P34)

More information about workflow in the x‐ray department. (P38)

## Discussion

This study is the first of its kind at the research site and therefore, generated new knowledge pertaining to the effectiveness of the CP programme offered to first‐year DR students. At the time of developing the CP programme, the authors found limited literature relating to CP programmes in DR and therefore, developed a self‐composed, three‐day face‐to‐face CP programme to facilitate the transition of first‐year DR students into the clinical environment.

The results of this study indicated that students experienced specific aspects of the CP programme interesting and beneficial to their preparedness for the workplace. Reinforcing and revising different types of injuries, watching radiographic positioning videos, performing practical positioning activities, and gaining knowledge of the various departments in the hospital environment were highlighted as the strengths of the CP programme. However, noteworthy changes were identified, which required a change to the structure and the content of the CP programme.

Students were of the opinion that the CP programme exhibited an excessive amount of theoretical content resulting in diminished concentration. Chen et al.^14^ concurred that content overload leads to a high cognitive burden and suggested that effective instructional strategies and only content relevant to its intended purpose, should be considered when designing educational programmes. The same authors proposed curricular and stakeholder input to provide guidance as to the breadth and depth of the content to be included to prepare students for their intended purpose. Rajab^15^ highlights an interdependence between the workplace, educational institutions and students to ensure a balance between the curriculum and workplace priorities. Therefore, and as key stakeholders, feedback from the students and workplace personnel would contribute significantly to the learning outcomes and the effective development of a similar CP programme.

Students indicated a need for the CP programme to focus more on simulation‐based learning (SBL) when they explained the need to be engaged in more practical learning relevant to the clinical environment. With SBL, students can acquire clinical skills necessary for the workplace in a safe learning environment such as the skills laboratory without compromising patient care.^16^ Allocating sufficient time to SBL in the CP programme is thus critical to enable students to gain the necessary confidence and competence in radiographic skills prior to commencing learning in the workplace.^17^ Learning through role play seems to be highly favoured by students as they reported that engaging in and acting out scenarios is an enjoyable and fun way to prepare them for the clinical environment. The literature in education and healthcare describes role play as beneficial to develop critical thinking as scenarios may demonstrate the real‐life situations students may encounter in the workplace. Furthermore, role‐play provides students with the opportunity to interact with their peers and facilitators, promote knowledge and kinesthetic learning, develop a professional identity, and improve communication and motivation.^18^, ^19^, ^20^ These attributes are all critical for functioning in the DR environment and should therefore be prioritised when the CP programme design is considered.

Findings from the literature reported that radiography students often feel unprepared for commencing placement in the clinical environment.^6^, ^21^ They often feel completely overwhelmed and experience uncertainty, anxiety, and stress when they transition from the classroom to the clinical environment. Research relating to nursing students' preparedness reported similar results when nursing students described a reality shock of the profession and fear of the unknown when commencing clinical for the first time.^22^ These findings contrast with the findings of the current study since students in this study reported feeling less anxious and felt better prepared for what is expected from them as first‐year students following the dedicated CP programme. They described feeling mentally and physically ready for the workflow, experienced less anxiety and felt prepared to act in emergency situations.

An awareness of the different equipment types and a more comprehensive focus on diverse patient needs were highlighted as a requisite for the CP programme. Students conveyed a superficial understanding of the various patient care needs and felt less prepared for interactions with patients who deviate from able patients. The notion of first‐year students not feeling prepared enough for different patient group interactions was supported by results from a similar study conducted in the United Kingdom.^21^ The various types of patients identified from the study included: the very ill, the badly injured, oncology patients, patients with dementia and unconscious patients. Although the present study does not refer to specific patient interactions, the preceding patient groups ought to be considered for inclusion into CP programmes. Specifically, because first‐year students' clinical rotation predominantly includes the general x‐ray department, orthopaedic department, the chest room, and trauma/emergency department where the likelihood of encountering such a patient is inevitable.

## Limitations

This study only considers the input from a single cohort. Future research endeavours should consider a longitudinal study to present a more holistic account of the student's readiness which may provide valuable feedback for incorporation into similar CP programmes. In addition, obtaining input from qualified radiographers in clinical practice and exploring their perceptions of the first‐year DR students' readiness upon entering the clinical environment is recommended.

## Conclusion and Implications for Practice

Due to the ever‐changing and evolving landscape associated with the delivery of healthcare services worldwide, effective CP programmes are critical for the successful transition of first‐year students from the classroom into the clinical environment. Healthcare educationalists should pay careful attention to and adapt to the needs and challenges of their students and the profession itself, by constantly reflecting on their pedagogic practices and by introducing innovative teaching and learning interventions. From the results of this study, it is clear that a balanced CP programme is important. This can be achieved by including more practical exposure, as opposed to theory, when preparing first‐year students for the workplace.

The collaborative development of an information pack between the first‐year lecturers and the clinical sites detailing the hospital layout, a staff organogram and contact persons will ease students' navigation. A pre‐clinical facility visit at the students' primary clinical site before official placement, also highlighted several benefits related to the familiarisation of one's new surroundings and witnessing first‐hand, the daily operational activities and workflow of the workplace. Although the current study sought to explore the perceptions of first‐year DR students only, the authors are of the opinion that the recommendations provided can be transferred and used to also inform the curricula of other healthcare professions wanting to adopt a similar approach.

## Funding Information

None.

## Conflict of Interest

The authors declare no conflict of interest.

## Data Availability

The data that support the findings of this study are available from the corresponding author upon reasonable request.
